# NGPINT V3: a containerized orchestration Python software for discovery of next-generation protein–protein interactions

**DOI:** 10.1093/bioinformatics/btaf343

**Published:** 2025-06-24

**Authors:** Schuyler D Smith, Valeria Velásquez-Zapata, Roger P Wise

**Affiliations:** Department of Plant Pathology, Entomology, and Microbiology, Iowa State University, Ames, IA 50011, United States; Oak Ridge Institute for Science and Education (ORISE), Oak Ridge, TN 37831, United States; Department of Plant Pathology, Entomology, and Microbiology, Iowa State University, Ames, IA 50011, United States; Oak Ridge Institute for Science and Education (ORISE), Oak Ridge, TN 37831, United States; Department of Plant Pathology, Entomology, and Microbiology, Iowa State University, Ames, IA 50011, United States; USDA-Agricultural Research Service, Corn Insects and Crop Genetics Research Unit, Ames, IA 50011, United States

## Abstract

**Summary:**

Batch yeast two-hybrid (Y2H) assays, leveraged with next-generation sequencing, have afforded successful innovations for the analysis of protein–protein interactions. NGPINT is a Conda-based software designed to process the millions of raw sequencing reads resulting from Y2H–next-generation interaction screens. Over time, increasing compatibility and dependency issues have prevented clean NGPINT installation and operation. A system-wide update was essential to continue effective use with its companion software, Y2H-SCORES. We present NGPINT V3, a containerized implementation built with both Singularity and Docker, allowing accessibility across virtually any operating system and computing environment.

**Availability and implementation:**

This update includes streamlined dependencies and container images hosted on Sylabs (https://cloud.sylabs.io/library/schuyler/ngpint/ngpint) and Dockerhub (https://hub.docker.com/r/schuylerds/ngpint), facilitating easier adoption and integration into high-throughput and cloud-computing workflows. Full instructions and software can be also found in the GitHub repository https://github.com/Wiselab2/NGPINT_V3 and Zenodo https://doi.org/10.5281/zenodo.15256036.

## 1 Introduction

Yeast two-hybrid (Y2H) is a powerful molecular technique to mine protein–protein interactions (PPIs) (Vidal and Fields 2014). In this method two fusion proteins are expressed in yeast, one protein of interest (the “bait”) is fused to the DNA-binding domain (DBD) of a transcription factor and another protein (the “prey”) to the activation domain (AD). If the bait and prey proteins interact, the DBD and AD are brought into proximity, reconstituting transcription and activating a reporter gene. Typically, activation of the reporter gene enables yeast growth under selection, indicating a positive interaction between bait and prey. Novel PPIs are critical for understanding complex biological processes and networks. For instance, in crop plant research, identifying interactions between plant proteins and pathogen effectors can lead to new insights into plant immunity and inform breeding strategies aimed at enhancing disease resistance. In biomedical research, PPIs drive drug target nomination and mode of action studies.

In order to improve the throughput and extract a higher number of significant interactions per assay, Y2H has been coupled with next-generation sequencing (NGS); the integration of these methods has been termed next-generation interaction screening (NGIS) (Suter *et al.* 2015, Pashkova *et al.* 2016, Yachie *et al.* 2016, Trigg *et al.* 2017, Erffelinck *et al.* 2018, Velásquez-Zapata *et al.* 2021, Elmore *et al.* 2023). In this implementation, NGS is used to quantify a library of preys that is screened against one or multiple baits, using prey quantification as the main variable to identify true PPIs. NGPINT is a python-based pipeline implementation of several bioinformatic software to process Y2H-NGIS data. With its companion software Y2H-SCORES, it is possible to identify hundreds of candidate PPIs from Y2H-NGIS experiments (Banerjee *et al.* 2021, Velásquez-Zapata *et al.* 2021, Velásquez-Zapata *et al.* 2023). NGPINT quantifies total prey abundancies in the Y2H-NGIS raw data. Additionally, the software characterizes Y2H interaction fragments by locating and quantifying the fusion reads between the prey vector and the prey sequence that interacts with the bait. Outputs from NGPINT include total and fusion counts, fusion read location, and mapped reads to the prey sequences to allow for prey characterization in Integrative Genomics Viewer (IGV) (Robinson *et al.* 2011). Output count data feed into Y2H-SCORES, which uses appropriate normalization methods, count data and statistical models to build a set of ranking scores to predict high-confidence PPI (Velásquez-Zapata *et al.* 2021).

As NGIS advances, appropriate software to process the complex datasets is crucial for the successful identification of PPI. NGPINT is fully adapted to this type of data—it identifies the sequences of interacting prey fragments and quantifies their abundance. By providing a reference genome, NGS data in fastq format, or optionally, a transcriptome reference, NGPINT can process data from any organism. The novelty of NGPINT to PPI studies is that its algorithms process Y2H-NGIS data, and particularly, detect fusion reads between the prey and the vector with high confidence, enabling the accurate identification of prey interaction fragments for cloning and orthogonal confirmation. This allows essential data to be recovered from NGIS sets, and therefore more complete functional interaction networks. In practice, maintaining the NGPINT software had been a challenging process as the Conda environment it was originally built on had become obsolete. Ensuring reproducibility and reliability for all scientific software is essential for the value of NGPINT, and of Y2H-SCORES. Here we accomplish this through containerization and present a new implementation of NGPINT built in both Singularity and Docker, designated NGPINT V3.

## 2 Results

### 2.1 Software framework

NGPINT orchestrates a diverse set of software. A common issue with these types of programs, ones having dependencies on third-party development and maintenance, is compatibility. NGPINT has not been an exception to these difficulties. After its initial development, despite providing an important scientific resource, NGPINT’s adoption into standard procedures for PPI analyses has been hindered by its availability. Using Conda environments for bioinformatics software comes with challenges such as complex dependency management, reproducibility issues, environment isolation, and cross-platform compatibility. Over time, as updates occur and clusters evolve, these issues can become problematic. In the NGPINT case, the Conda environment for V1 required the installation of >200 individual accessory packages. This, in turn, created dependency conflicts when updates in some of the individual packages became necessary. Eventually, installation of the software became impractical given the unavailability/incompatibility among packages. This made reproducibility difficult, especially as the exact versions of packages used for the original development were no longer available, or if newer versions introduced incompatibilities. Examples include software such as samtools which changed from V7 to V14, R which changed from V3 to V4, and multiple GCC dependencies across multiple packages. To address these issues, Docker and Singularity containers were used to improve package management of the entire runtime environment, ensuring software consistency across different systems and simplifying deployment.

**Figure 1.**
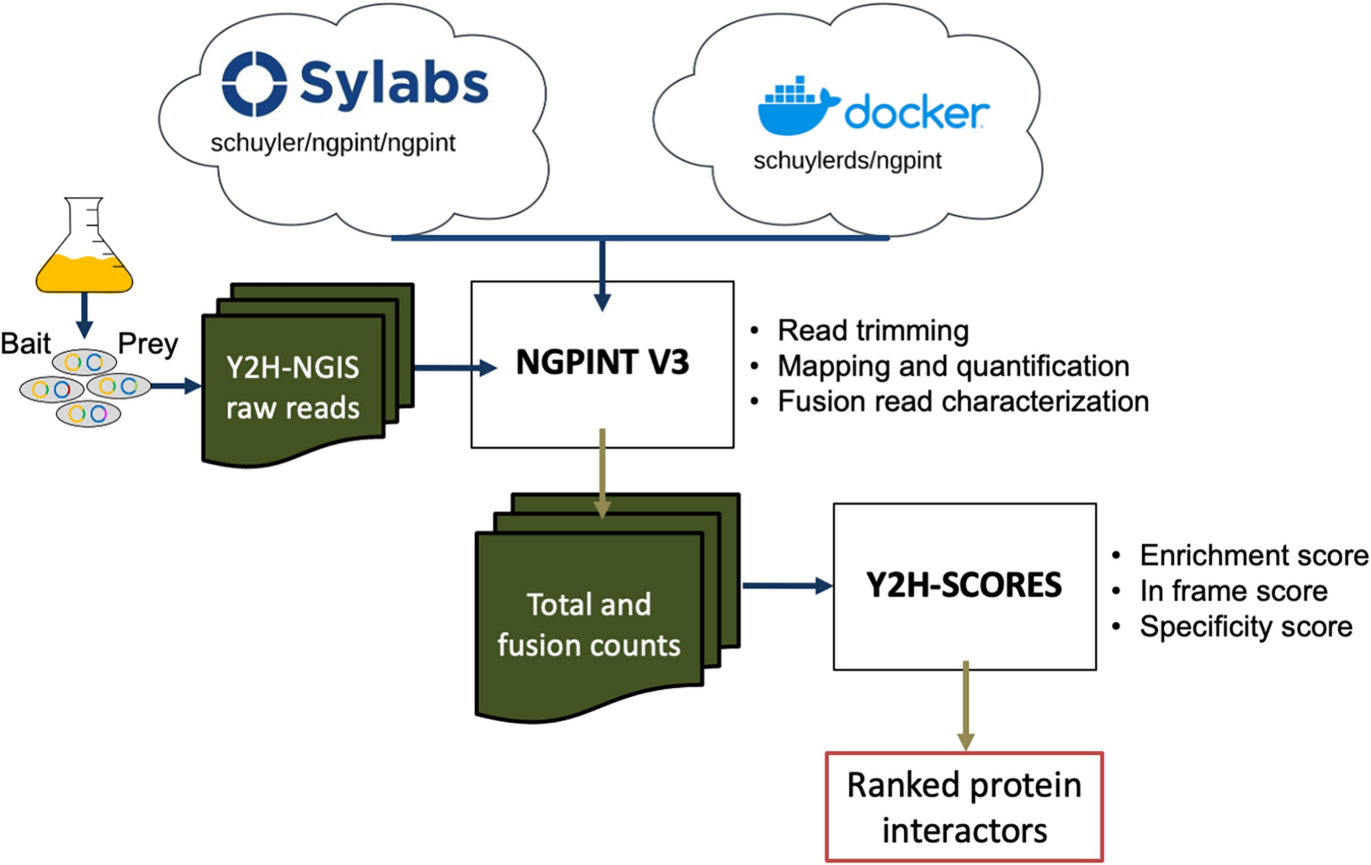
NGPINT V3. NGPINT V3 has been constructed into container images hosted on both Sylabs Singularity Container Services and Dockerhub, as well as a dockerfile. NGPINT V3 provides a mutable set of instructions to reconstruct or edit the workable environment. These containers ensure easy installation of the NGPINT software. Y2H-SCORES uses the NGPINT outputs to generate a ranked list of bait/prey interactions.

As shown in Fig. 1, in this release we have updated the dependencies required, trimmed down as many unnecessary or redundant dependencies as possible, and constructed container images hosted on both Sylabs Singularity Container Services and Dockerhub, as well as a dockerfile within the NGPINT V3 repository to provide a mutable set of instructions to reconstruct or edit the workable environment. Providing prebuilt containers for NGPINT solves two of the largest usability issues for the software; (i) it provides a method that does not require additional installation or setup, and (ii) it allows the software to be used universally across any operating system, where it originally was only able to run on Unix-based machines. This version of the software should provide a more user-friendly experience adopting NGPINT into workflow and provide out-of-the-box availability for high-throughput computing clusters and cloud-computing services.

### 2.2 Illustrative example

The containerized versions of the software are designed to use the program with the same commands and arguments documented and outlined as the original (V1) software from the command-line. The main difference is the use of the container orchestration software and the elimination of conflicting dependencies, as they are installed into the containers in static, isolated, environments in Docker or Singularity. The updated code and running instructions for the software can be found on the GitHub page https://github.com/Wiselab2/NGPINT_V3. Both Docker and Singularity are technologies for containerization of software, however Singularity is designed primarily for use in high-performance computing (HPC) environments such as university clusters. Both methods require installation of the container software (Docker/Singularity) to use the containers. Often, Singularity will be installed on clusters by the administrators, Docker is typically not feasible to run in an HPC environment. If there is not a specific reason to be using Docker, Singularity would be the preferred choice for most scientific computing.

The toy dataset from NGPINT V3 is a simulated *Arabidopsis thaliana* Y2H-NGIS dataset with one bait, two conditions (selected and non-selected or background) and three replicates. NGS reads were generated using an RNA-Seq simulator called Polyester (Frazee *et al.* 2015) by setting differentially enriched transcripts between selected and non-selected samples as true positives. Inputs to NGPINT V3 are indicated in a configuration csv file which contains the full paths to the files (Fig. 1, available as supplementary data at *Bioinformatics* online). The pipeline requires sequencing data in fastq format, a reference genome or transcriptome in FASTA format, along with an annotation file in gene transfer format. Additionally, the configuration file must include a FASTA file containing the plasmid sequences for both bait and prey. Full instructions to run the software can be found in the GitHub repository https://github.com/Wiselab2/NGPINT_V3 and Zenodo https://doi.org/10.5281/zenodo.15256036.

## 3 Discussion

NGPINT presents a tool for significant advancement in the field of PPI research, particularly for studies utilizing next-generation sequencing coupled with Y2H screening. By addressing the major usability issues associated with dependency management and software compatibility, NGPINT V3 enhances both accessibility and reliability. The implementation of Docker and Singularity containers facilitates integration into the most common computing environments in scientific research, including high-throughput and cloud-based systems, broadening the software’s usability for researchers across all Unix and Windows based platforms.

By providing a version that eliminates the need for an installation process and coordinating dependency conflicts, we have built a more user-friendly experience that we hope will promote wider adoption and consistent application of the value provided in NGPINT. This enhanced accessibility is expected to accelerate PPI research by enabling more researchers to efficiently analyze large-scale Y2H-NGIS datasets. Furthermore, the ability to process data from any organism with an available reference genome means that NGPINT V3 can be applied to a wide range of biological contexts, from model organisms to other species.

The improvements in NGPINT V3 facilitate the discovery of novel PPIs, as accessible Y2H-NGIS software is required for accurate identification of PPIs at large scale. The initial development of NGPINT and Y2H-SCORES addressed a key challenge in Y2H-NGIS analysis by designing customized software with robust statistical frameworks to ensure reliable data interpretation. Maintaining the reproducibility of these tools over time is essential for sustaining meaningful PPI discoveries and advancing interaction screening methodologies. The broader impact of NGPINT V3 extends to any field where high-throughput PPI studies are relevant, contributing to advancements in molecular biology, genomics, and systems biology.

## Supplementary Material

btaf343_Supplementary_Data

## Data Availability

All data and code are available in the GitHub page https://github.com/Wiselab2/NGPINT_V3 and Zenodo DOI https://doi.org/10.5281/zenodo.15256036. Additionally, the software was submitted to the containers in Sylabs https://cloud.sylabs.io/library/schuyler/ngpint/ngpint and Dockerhub https://hub.docker.com/r/schuylerds/ngpint.
