# Supplementary material for: NGPINT V3: a containerized orchestration Python software for discovery of next-generation protein–protein interactions
Source: Bioinformatics. 2025 Jun 24;41(6):btaf343. doi: 10.1093/bioinformatics/btaf343 (PMC12208055; doi:10.1093/bioinformatics/btaf343)
Supplement: btaf343_Supplementary_Data [file btaf343_supplementary_data.zip › Smith Supplemental Fig 1.pdf]

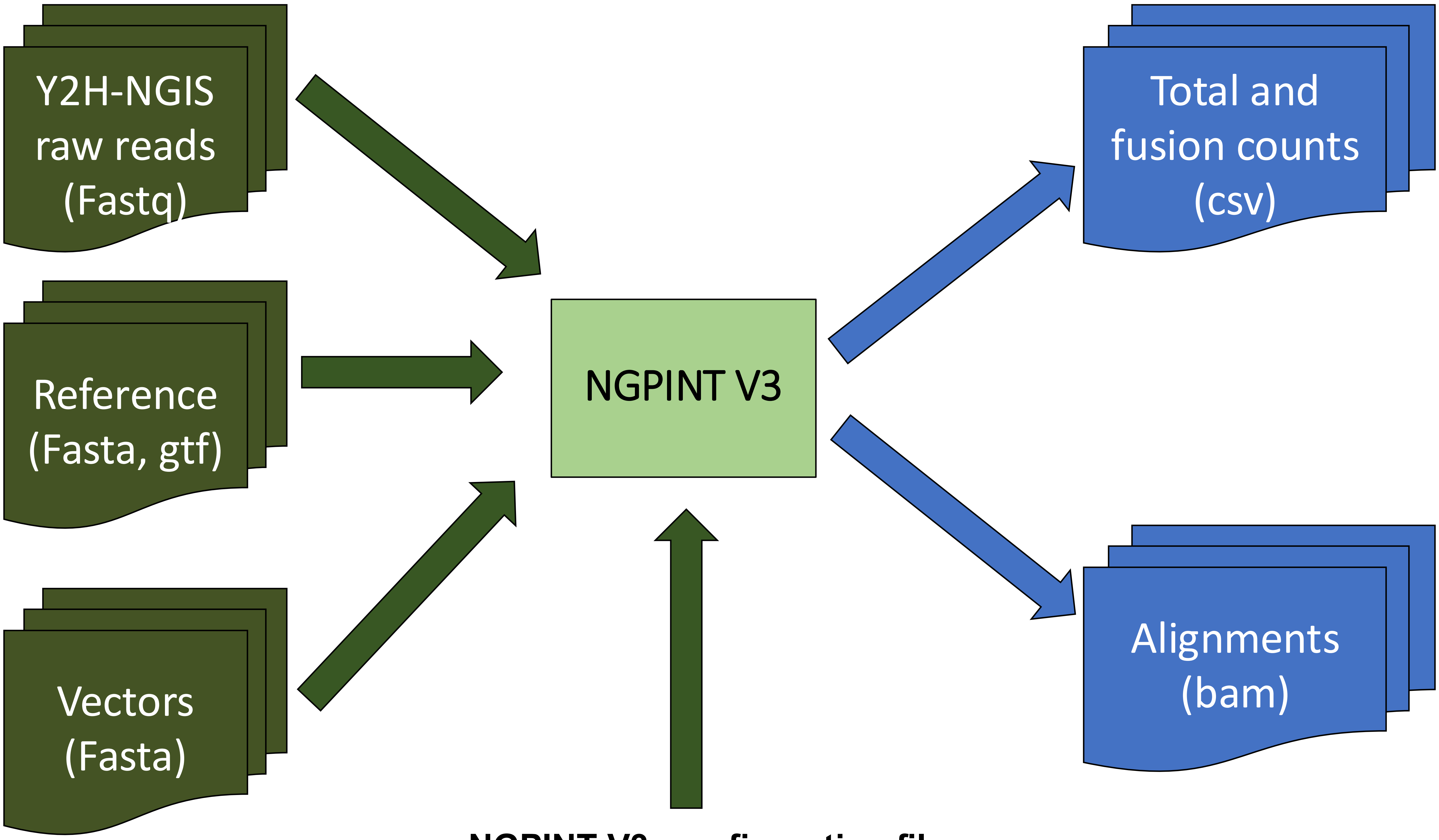

NGPINT V3 configuration file

Argument name	Argument description	Required/ Optional	Default Argument value	Argument value
input_ended	Single-end (SE) or Paired-end (PE)	1	SE	
input_fullpath	Path to S fastq files	1	None	
background_ended	Single-end (SE) or Paired-end (PE)	1	SE	
background_fullpath	Path to NS fastq files	1	None	
genome	Path to ref genome	1	None	
star_genome_index	Path to star gen index	0	None	
output_directory	Path to output	1	None	
plasmid_sequences	Path to plasmid fasta	1	None	
five_prime_vector	Seq of the 5' vector	1	None	
three_prime_vector	Seq of the 3' vector	1	None	
CPU	# of CPUs	0	1	
frame_of_TF_fusion	# bases to make prey fusion *in-frame*	0	0	
nucleotide_for_frame_shift	Base used for frame-shift	0	None	
min_trimmed_length	Min seq left after trimming	1	15	
force	Overwrites the output directory	1	0	
clean_up	Delete temp files	0	0	
gtf_annotation	Path to GTF file	1	None	
functional_annotation	Path to annotation	0	None	
transcriptome_index	Path to star transcriptome index	1	None	

Supplementary Figure 1. List of inputs and outputs for the NGPINT V3 pipeline.
